# Crosstalk Between Cancer-associated Fibroblasts and Myeloid Cells Shapes the Heterogeneous Microenvironment of Gastric Cancer

**DOI:** 10.2174/0113892029300608240531111743

**Published:** 2024-06-11

**Authors:** Zhiwei Peng, Can Fang, Zhiwei Tong, Qiufan Rao, Zihao Ren, Kongwang Hu

**Affiliations:** 1 Department of General Surgery, First Affiliated Hospital of Anhui Medical University, Hefei, Anhui 230022, China;; 2 Anhui Province Key Laboratory of Major Autoimmune Diseases, Anhui Institute of Innovative Drugs, School of Pharmacy, Anhui Medical University, Hefei, Anhui 230032, China;; 3 Department of General Surgery, Fuyang Affiliated Hospital of Anhui Medical University, Fuyang, Anhui 236000, China

**Keywords:** Single-cell sequencing, gastric cancer, cancer-associated fibroblasts, myeloid cells, tumor microenvironment, ligand-receptor pairs

## Abstract

**Background:**

Targeted therapies have improved the clinical outcomes of most patients with cancer. However, the heterogeneity of gastric cancer remains a major hurdle for precision treatment. Further investigations into tumor microenvironment heterogeneity are required to resolve these problems.

**Methods:**

In this study, bioinformatic analyses, including metabolism analysis, pathway enrichment, differentiation trajectory inference, regulatory network construction, and survival analysis, were applied to gain a comprehensive understanding of tumor microenvironment biology within gastric cancer using single-cell RNA-seq and public datasets and experiments were carried out to confirm the conclusions of these analyses.

**Results:**

We profiled heterogeneous single-cell atlases and identified eight cell populations with differential expression patterns. We identified two cancer-associated fibroblasts (CAFs) subtypes, with particular emphasis on the role of inflammatory cancer-associated fibroblasts (iCAFs) in EMT and lipid metabolic crosstalk within the tumor microenvironment. Notably, we detected two differentiation states of iCAFs that existed in different tissues with discrepant expression of genes involved in immuno-inflammation or ECM remodeling. Moreover, investigation of tumor-infiltrating myeloid cells has revealed the functional diversity of myeloid cell lineages in gastric cancer. Of which a proliferative cell lineage named C1QC^+^MKI67^+^TAMs was recognized with high immunosuppressive capacities, suggesting it has immune suppression and cell proliferation functions in the tumor niche. Finally, we explored regulatory networks based on ligand-receptor pairs and found crucial pro-tumor crosstalk between CAFs and myeloid cells in the tumor microenvironment (TME).

**Conclusion:**

These findings provide insights for future cancer treatments and drug discovery.

## 1 INTRODUCTION

Gastric cancer (GC) is one of the most lethal malignant tumors originating from the gastric mucosa and has a poor prognosis. Currently, GC remains common cancer with a particularly high incidence in Eastern Asia and was responsible for over 1 million new cases and more than 760,000 deaths worldwide in 2020, ranking fifth in incidence and fourth in mortality among known cancers [1]. Substantial progress has been made in identifying crucial tumor driver genes and performing precision treatments targeting these molecules in many cancer types, including breast and colorectal cancers [2]. However, improvements in the clinical outcomes of GC remain modest owing to the extensive intra-patient and intra-tumor heterogeneity of the tumor microenvironment (TME) [2].

The TME is a complex system comprising various cellular and acellular components, including endothelial cells, fibroblasts, vessels, and immune cells [3, 4]. Over the past few years, the tumor microenvironment has been widely investigated for multiple tumor types in cancer biology. The extensive interactions between various extracellular components and cancer cells in the TME play important roles in tumor development, metastasis, and chemoresistance [3, 5]. Cancer-associated fibroblasts (CAFs), the main stromal cells, can promote tumor progression through crosstalk with complex stromal components *via* extracellular matrix (ECM) remodeling and secretion of multiple growth factors, cytokines, and chemokines [6-8]. In addition, tumor regions contain abundant tumor-infiltrating myeloid cells, including tumor-associated macrophages (TAMs), dendritic cells (DCs), and monocytes, which exert tumor-promoting or-suppressive roles in the TME through reciprocal interactions with CAFs [3, 9-11]. However, the tumor promotion or suppression mechanisms and the interactions of CAF-myeloid cells are still insufficient owing to limited technology. Recently, single-cell sequencing has been a rapidly developing technique that may revolutionize our appreciation of the biological features and dynamics within CAFs and myeloid cells in the tumor microenvironment at single-cell resolution [12]. Therefore, the integration of high-throughput single-cell sequencing technology and experimental methods might provide insights to decipher the mechanisms of TME in more detail for precision medicine [13].

In this study, we analyzed the single-cell transcriptomes of 12 samples and profiled the atlases of TME heterogeneity from primary gastric tumors, para-tumor tissues, and metastases across 31017 single cells. Using bioinformatics analysis and experimental validation, we highlighted the comprehensive roles of CAFs and tumor-infiltrating myeloid cells in GC. Using trajectory analysis, we identified the differentiation trajectories of CAFs and myeloid cells, characterizing the functional heterogeneity of the diverse cell lineage states. In addition, the CAF-myeloid cell regulatory network established by Cellchat [14] profiles novel pro-tumor interactions between CAFs and myeloid cells in the TME. In addition, the clinical prognosis of specific cell types was examined using the public TCGA dataset. These findings not only broaden our understanding of the biological characteristics of TME components in GC but also provide potential precise targets for GC treatment.

## 2 MATERIALS AND METHODS

### 2.1 Dataset Source

Single-cell RNA-seq data utilized in our project were downloaded from the Gene Expression Omnibus (GEO: GSE183904) repository at https://www.ncbi.nlm.nih.gov/geo/query/acc.cgi?acc=GSE183904. The single-cell dataset was first examined using qualified data, and 12 samples were reanalyzed: five samples were derived from primary gastric tumor tissues, four from adjacent normal gastric tissues, and three from peritoneal metastases [15].

### 2.2 Single-cell Sequencing Processing

The R package Seurat (v4.1.2) was used to process single-cell RNA-seq data [16]. Cells containing 500 or more features and less than 4000 features were considered for subsequent analysis, and cells with mitochondrial RNA percentages greater than 15% were filtered out in this study. The SCTransform method was used to normalize the data. The functions SelectIntegrationFeatures, PrepSCTIntegration, FindIntegrationAnchors, and IntegrateData were used to remove batch effects and integrate different Seurat objects into a single dataset. To reduce the dimensionality of the data, the integrated data were subjected to principal component analysis (PCA), following FindNeighbors and FindClusters, which were used to cluster similar cellular populations. Data visualization was performed using Uniform Manifold Approximation and Projection (UMAP). Cells were annotated by combining the SingleR tool (v1.4.1, dataset: HumanPrimaryCellAtlasData) and marker genes. CellCycleScoring function was applied to infer the cell cycle by scoring the cell cycle marker (geneset: cc.genes) of single cells.

### 2.3 Differential Gene Expression and Enrichment Analysis

Differentially expressed genes (DEGs) of distinct cellular populations were identified using the FindAllMarkers module, and positively expressed genes and genes expressed in more than 25% of the cells in each cluster were selected. To analyze the immunosuppression and EMT scores of each cell [15, 17, 18], we performed single-sample gene set enrichment analysis (ssGSEA) on the expression matrix of single cells using GSVA (v1.38.2) [19, 20]; pathway enrichment was based on the average expression matrix of clusters. We set *p* <0.05, and |avg. log FC | >1 as the cutoff criterion.

### 2.4 Single-cell Metabolism Activity Scores Using scMetabolism

We utilized the newly developed R package scMetabolism (v0.2.1) to quantify single-cell metabolic activity, the robustness for single-cell data of which has been proven [21], the VISION method [22], and KEGG metabolic gene sets were applied to calculate the metabolic activity of each cell. DotPlot.metabolism and BoxPlot.metabolism functions were used to visualize the metabolic patterns of different cell types.

### 2.5 Trajectory Analysis

A pseudotime analysis was conducted to evaluate cell differentiation in the TME to explore the transitional relationships among different cellular populations using monocle2 (v2.18.0) [23]. The plot_genes_in_pseudotime function was used to visualize changes in gene expression among the different clusters. BEAM analysis was used to determine the pivotal roles of a series of genes in cellular differentiation. The plot_genes_branched_heatmap and plot_genes_ branched_pseudotime modules were used to visualize the key gene expression trajectories during differentiation. We set qval < 0.0001 as the cutoff value.

### 2.6 Cell-cell Communication with Cellchat

Cellchat (v1.1.3) is a new computational method developed by Jin *et al.* [14] that can infer the cell-cell communication atlas between single cells and build a regulatory network based on ligand-receptor crosstalk. Fibroblast and myeloid cell clusters identified in our dataset were investigated using Cellchat to explore communication networks; we selected significantly changed ligand-receptor pairs and used the netVisual function to visualize the results. To further analyze the major signals for specific cell subgroups and general communication patterns, we applied non-negative matrix factorization (NMF) algorithms, and the k value was selected as three. The identifyCommunicationPatterns function was then used to calculate the incoming communication patterns, and the netAnalysis_river function was applied to visualization.

### 2.7 Correlation to TCGA Dataset Cohort

To confirm the clinical prognosis of specific cells discerned by the single-cell dataset, the TCGA STAD cohort transcriptome dataset was downloaded from UCSC Xena: https://xenabrowser.net/datapages/. We selected the top 20 DEGs as specific cell type genes and scored them by GSVA; then, the median was used as the cutoff value to stratify patients into distinct groups (high or low proportion). Survival analysis was performed using the R package survminer (v0.4.9). A log-rank test was applied to calculate the statistical significance of patients stratified into different groups.

### 2.8 Immunofluorescent Staining

An immunofluorescence (IF) assay was conducted to confirm the presence of specific myeloid cell sublineages identified in our study. The following antibodies were used to detect cell-specific protein expression in distinct myeloid cells: anti-osteopontin (rabbit, 1:200, HuaBio, ER1802-16) and anti-CD68 (mouse, 1:200, HuaBio, EM1706-11). Frozen sections from GC tissues or adjacent normal tissues (n=2 pairs) were pre-treated with heat antigen retrieval (TRIS-EDTA, pH8) for 15 min. Tissues were blocked with 5% BSA for 40 min. Sections were incubated with antibodies (described above) separately at 4°C overnight. After washing thrice with PBS, goat anti-rabbit IgG antibody (488, HA1121) and goat anti-mouse IgG antibody (555, HA1118) were probed at a 1:600 dilution for 1 h. Finally, we used DAPI to stain the cell nucleus. The tissue autofluorescence was eliminated using a tissue autofluorescence removal kit (Servicebio, G1221).

### 2.9 Multiplex Immunohistochemistry

The multiplex immunohistochemistry (mIHC) method was used to detect three different antibodies in the sections. The primary antibodies include C1QC (Rabbit, Cat. No. BS-11337R; Bioss, 1:3000), anti-Ki67 (Rabbit, 1:500; HuaBio, Cat. No. HA721115), and CD68 (Rabbit, Cat. No. EM1706-11, HuaBio, 1:500). Cancer tissues or adjacent normal tissues used for the mIHC experiment were obtained from previously collected paraffin-embedded surgical specimens from patients with GC who signed informed consent forms. A TSAPLus Fluorescence Triple Staining Kit (Cat. No. G1236-50T, Servicebio) was used to stain the primary antibodies. On the first day, we repaired the antigen, inactivated endogenous peroxidases, blocked the antigen, and labeled the sections with the first primary antibody C1QC at 4°C overnight. On the second day, after labeling with 488-TSA (1:500), the tissue sections were treated with an antigenic repair solution. Further, we repeated the previous steps and incubated the sections with two primary antibodies, Ki67 and CD68, at 4°C overnight. On the third day, the sections were stained with CY3-(1:300) and CY5-(1:300) conjugated secondary antibodies for 50 min. Finally, we used DAPI to stain the nucleus. The tissue autofluorescence was eliminated using a tissue autofluorescence removal kit (Servicebio, G1221).

### 2.10 Immunohistochemical Method

Immunohistochemistry (IHC) was used to detect cell-specific protein expression in distinct GC fibroblasts. Antibodies, including anti-PDGFRA (Rabbit, 1:150, HuaBio, Cat. No. ET1702-49) and anti-RGS5 (rabbit, 1:150, Affinity, Cat. No. DF4417) were used for the IHC analysis. Paraffin-embedded GC or adjacent normal tissue sections (n=3 pairs) were treated with a boiling antigenic repair solution (TRIS-EDTA, pH9) for 15 min. Endogenous peroxidases were inactivated with 3% hydrogen peroxide, and the tissues were blocked by 5% BSA for 40 minutes. Primary antibodies were added in sections for incubation overnight at 4°C. The tissues were probed with an HRP-conjugated compact polymer system after washing with PBS the following day, and diaminobenzidine (DAB) was used as a chromogen. Finally, the nuclei were stained with hematoxylin. Double immunohistochemical staining for PDGFRA (rabbit, 1:150; HuaBio, Cat. No. ET1702-49) and CD163 (mouse, 1:150; HuaBio, Cat. No. EM1901-90) was performed according to the instructions of the immunohistochemical double staining kit (ZSGB-Bio, Cat. No. DS-0004) and horseradish peroxidase-conjugated secondary antibodies were mixed for 30 min at room temperature, and DAB and AP-red were used as chromogens.

### 2.11 Statistical Analysis and Visualization

All data analyses were based on R (v4.1.2), and data visualization was performed using the R packages Seurat (v4.0.2), pheatmap (v1.0.12), Cellchat (v1.1.3), scMetabolism (v0.2.1), monocle2 (v2.18.0), ggplot2 (v3.3.5), and Survminer (v0.4.9).

## 3 RESULTS

### 3.1 scRNA-seq Reveals the Tumor Microenvironment Diversity in Gastric Cancer

**Fig. (1):**
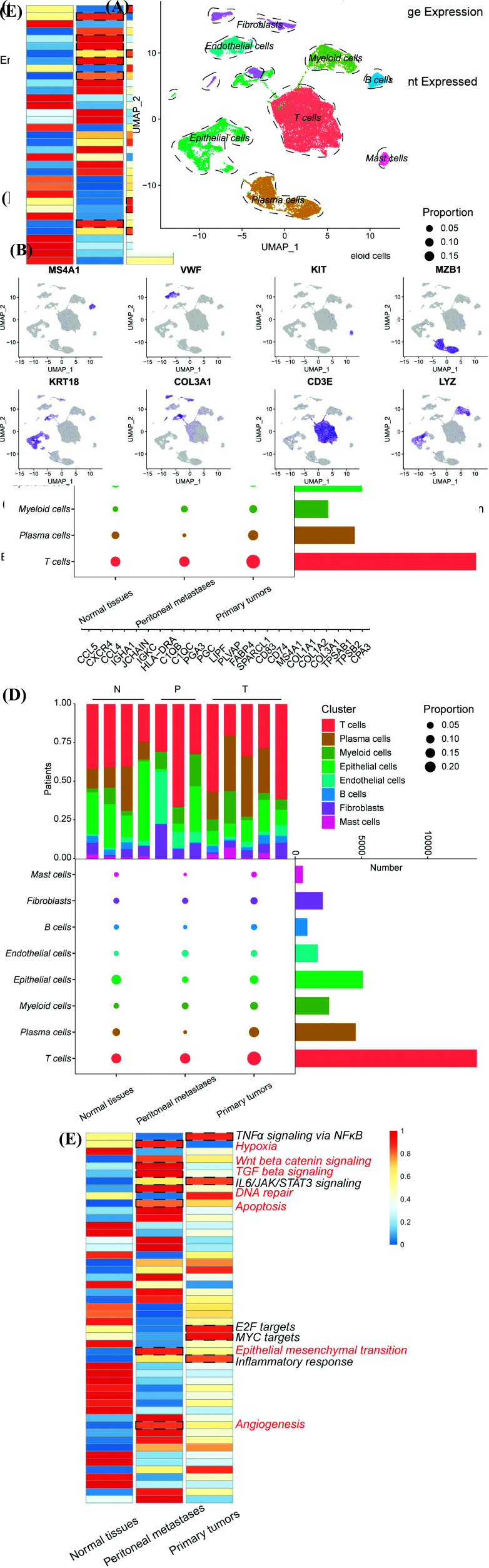
scRNA-seq reveals the tumor microenvironment diversity in gastric cancer. (**A**) UMAP plots of single cells RNA-seq profiled in this project colored by cell types. (**B**) Feature plots show the cell markers expression in each cell types. (**C**) Dot plot exhibits the top 3 differentially expressed genes (DEGs) of different cellular populations. (**D**) Cell proportions of different tissues or patients calculated by scRNA-seq dataset. (**E**) Heatmap shows ssGSEA result to explore the functional enrichment of different tissues including adjacent normal gastric tissues, peritoneal metastases and primary gastric tumors.

In this study, we reanalyzed single-cell sequencing data from a previously published study that included 12 samples (Patient 1–12) [15]. After quality control and removal of batch effects (Fig. **S1**), 31017 cells were included in this study. We applied PCA to reduce the dimensions and cluster the cells into eight different populations (Fig. **1A**). Based on the identified cluster-specific genes (Figs. **1B** and **C**), we combined SingleR (v1.4.1, dataset: HumanPrimaryCellAtlasData) [24] and canonical marker genes [17, 25-30] for cell cluster annotation: epithelial cells (KRT18+), endothelial cells (VWF+), T cells (CD3E+), myeloid cells (LYZ+); Fibroblasts (COL3A1+); Plasma cells (MZB1+); B cells (MS4A1+), and mast cells (KIT+) (Fig. **1B** and Table **S1** and **2**). Further, we analyzed the cell proportions of different tissues (Fig. **1D**). We found that immune cells such as T, plasma, and myeloid cells were present in high proportions in primary tumor tissues rather than those in metastases, indicating that immunoinflammatory responses existed in tumors. In contrast, the reduced number of plasma cells and B cells in metastases may be attributed to the immunosuppressive microenvironment (Fig. **1D**) [21, 31, 32]. To further explore the functions of distinct microenvironments, we calculated the average gene expression in different pathological tissues and conducted an enrichment analysis using ssGSEA (Fig. **1E** and Table **S3**). The results showed that the primary tumor microenvironment was significantly enriched with various tumor-related pathways, such as TNFα/NFκB-signaling and IL6-JAK-STAT3-signaling. In addition, the primary tumors showed a significant inflammatory response, which was consistent with the above analysis. However, metastatic cancer underwent other tumor-driver signaling, including the Wnt/β-catenin pathway and TGF-β signaling. In addition, malignant tumor phenotypes such as EMT progression, angiogenesis, hypoxia niche, and cell cycle-related pathways were also enriched in metastatic tumors, suggesting that these factors are related to the formation of a tumor metastasis microenvironment [33].

### 3.2 Two Different Types of Cancer-associated Fibroblasts are Identified in GC

**Fig. (2):**
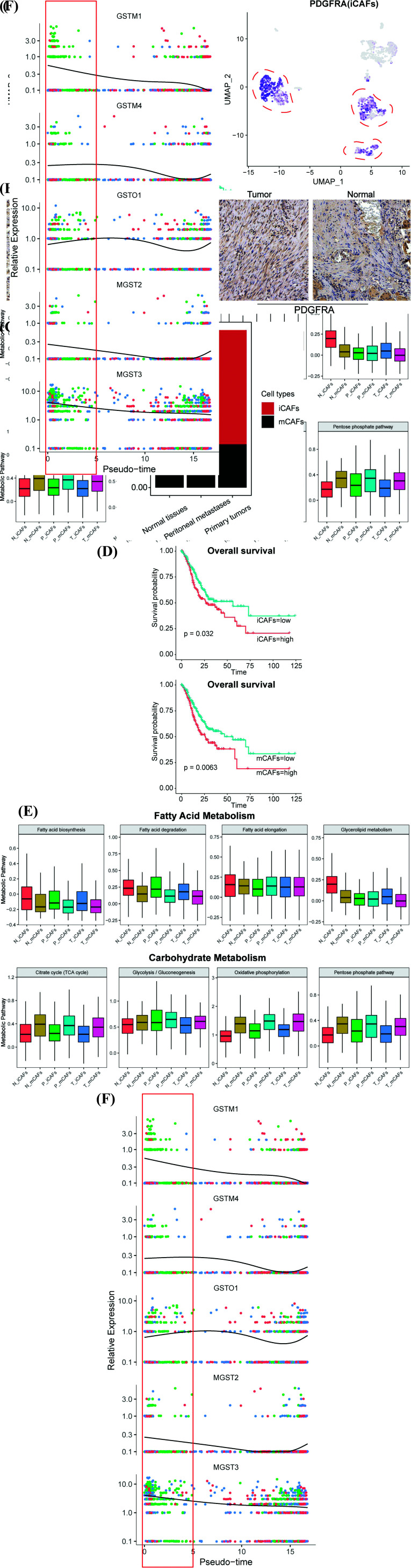
Two different types of cancer-associated fibroblasts are identified in GC. (**A**) Feature plots of fibroblasts colored by cell markers expression (RGS5, PDGFRA), the deeper the color, the higher the expression. (**B**) IHC analysis of PDGFRA and RGS5 expression show the existence of mCAFs or iCAFs in gastric cancer. (Scale bars: 50μm) (**C**) Bar plot shows the cell proportions of iCAFs and mCAFs in distinct tissues. (**D**) K-M plots show the high proportions of two types of CAFs result in poor clinical prognosis of patients with gastric cancer in TCGA STAD cohort, OS: overall survival. (**E**) Box plots show the differential expression patterns of fatty acid metabolism or carbohydrate metabolism between six subtypes of cancer-associated fibroblasts. (**F**) Key genes expression on CAFs-mediated drug metabolisms in metastases are inferred by pseudo-time trajectories, pink dots represent normal tissues, green dots represent peritoenal metastases and blue dots represent primary tumors.

To further explore the functional roles of stromal components in the microenvironment, fibroblasts were selected for subsequent studies. Reclustering of fibroblasts was identified into two subtypes based on classic gene markers: subcluster1, which highly expressed the inflammatory cancer- associated fibroblast cell marker (PDGFRA), was described as iCAFs and other subclusters expressing RGS5 related to myo-cancer-associated fibroblasts described in previous studies were named mCAFs (Fig. **2A**, Fig. **S2**, and Table **S4**) [29, 34, 35]. As shown in Fig. (**2B**), we confirmed that GC cells were enriched with iCAFs and mCAFs by immunohistochemical staining for PDGFRA and RGS5. In addition, we detected that mCAFs were highly enriched in metastases, which was speculated to be associated with the malignant behavior of the metastatic niche (Fig. **2C**). In addition, the TCGA STAD cohort was analyzed to explore the clinical significance of both subgroups in GC, and our results suggested that high proportions of iCAFs and mCAFs could result in poor clinical prognosis (Fig. **2D**).

To completely depict the heterogeneous metabolic landscape of CAFs in GC, CAFs were further classified into six subtypes: primary tumor CAFs (T_CAFs), normal tissue CAFs (N_CAFs), and peritoneal metastasis CAFs (P_CAFs), and scores of known metabolic pathways in each cell were computed using scMetabolism (Fig. **S3A** and Table **S5**) [21]. Emerging evidence has shown that CAFs can promote tumor metastasis through the lipid metabolic crosstalk with tumor cells or other stromal cells in TME [36, 37]. Therefore, we specifically examined lipid metabolism within six subtypes of CAFs, and our studies confirmed that iCAF subtypes have higher lipid metabolism levels than mCAFs, indicating the pivotal role of iCAFs in fatty acid metabolism in the TME, whereas mCAFs were largely related to carbohydrate metabolism, especially the TCA cycle and oxidative phosphorylation (Fig. **2E**). Furthermore, we also discovered that P_CAFs were closely related to drug metabolism (Fig. **S3B**). Pseudo-time analysis was conducted to identify key drug metabolic genes, which demonstrated that Glutathione S-transferase (GST) families mainly participated in CAF-mediated drug metabolism in metastases (Fig. **2F**), highlighting that these genes might be responsible for CAF-mediated chemoresistance formation in metastatic tissues of GC.

### 3.3 Pseudotime Analysis Profiles Differentiation Trajectories of Two Types of CAFs

**Fig. (3):**
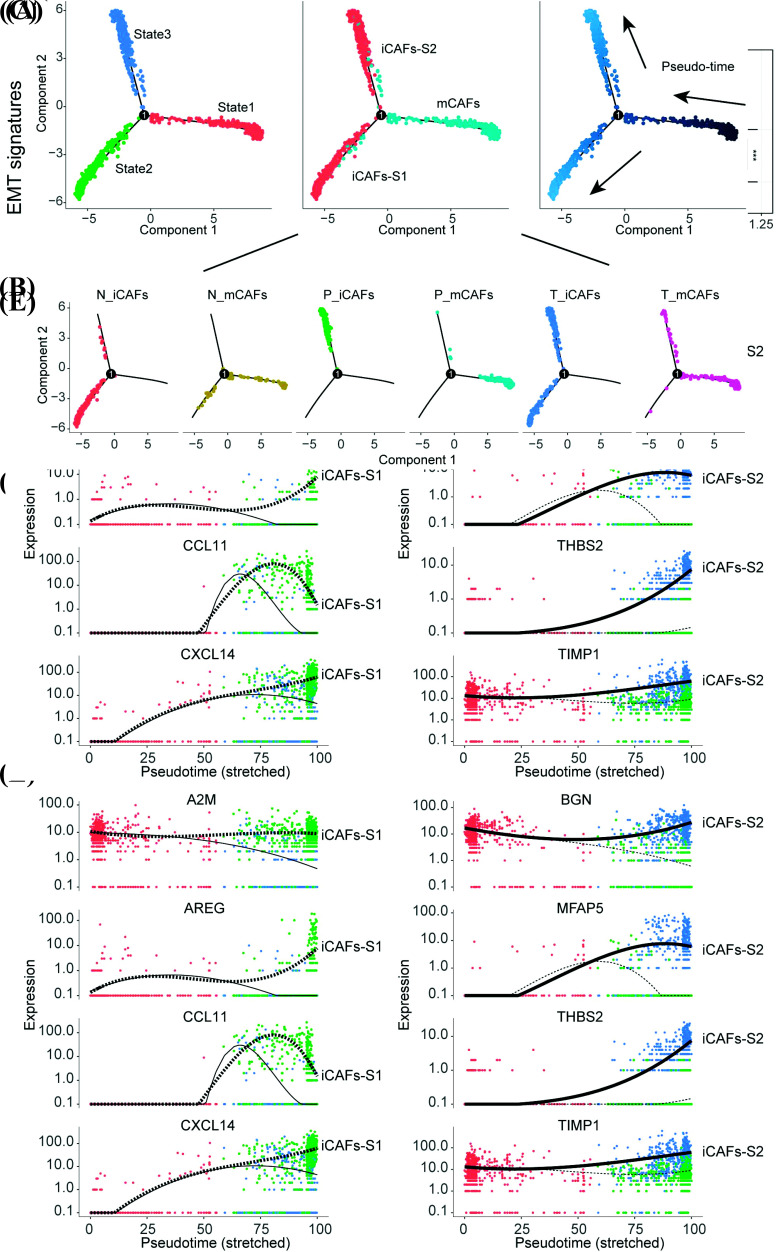
Pseudotime analysis profiles differentiation trajectories of two types of CAFs. (**A**) Differentiation trajectories of CAFs colored by state, cell subtypes and pseudo-time. (**B**) Differentiation trajectories of six CAFs subgroups. (**C**) Gene expression of EMT signatures in single cell ordered along the inferred pseudo-time. (**D**) Ridge plot of EMT scores in two distinct CAFs subtypes calculated by ssGSEA on known EMT gene markers (Methods). (**E**) Pseudotime plots of differential expression of genes involved in immuno-inflammation or ECM remodeling in distinct cell fates, pink dots represent State 1 (Fig. **3A** left), green dots represent State 2 (Fig. **3A** left) and blue dots represent State 3 (Fig. **3A** left). (branches: iCAFs-S1: State 2 and iCAFs-S2: State 3). ***: *p*<0.05

Recent studies have shown that CAFs are a heterogeneous population with numerous potential cellular sources that perform various functions [6]. Therefore, we applied pseudotime analysis to the two subtypes of CAFs, aiming to discover transitional changes in differentiation trajectories (Figs. **3A** and **B**). Li *et al.* previously confirmed that upregulated EMT signatures are driven by CAFs rather than tumor epithelial cells in colorectal cancer [28]. In our trajectory plots, we further discovered that iCAFs, not mCAFs, play a crucial role in EMT (Fig. **3C**). In addition, we calculated the EMT scores of CAFs based on 237 genes from Kumar *et al.* [15], and the results verified that iCAFs highly expressed EMT signatures (Fig. **3D** and Table **S6**). We first found that iCAFs could be divided into two states (S1–2) according to specific transcription patterns (Fig. **3A**, middle). iCAFs-S1 mainly existed in normal tissues, and iCAFs-S2 was mostly enriched in metastases, both of which can exist in primary tumors. BEAM analysis of the two states of iCAFs revealed that iCAFs-S1 highly expressed immunoinflammatory genes, whereas iCAFs-S2 were strongly associated with ECM remodeling (Fig. **3E** and Fig. **S4**). In particular, these highly expressed genes were notably associated with tumor development and metastatic progression, including A2M, AREG, chemokines (iCAFs-S1), BGN, MFAP5, THBS2, and TIMP1 (iCAFs-S2) (Fig. **3E** and Fig. **S4**) [38-43]. In general, we speculated that the diversity of the tissue microenvironment niche leads to tissue-specific functional differences in CAFs, which in turn could facilitate tumor development in different ways. Thus, tailored therapies targeting CAF-specific functional signatures may be optimal for carcinoma treatment.

### 3.4 Characterization of scRNA-seq Trace Diverse Myeloid Cell Lineages in GC

**Fig. (4):**
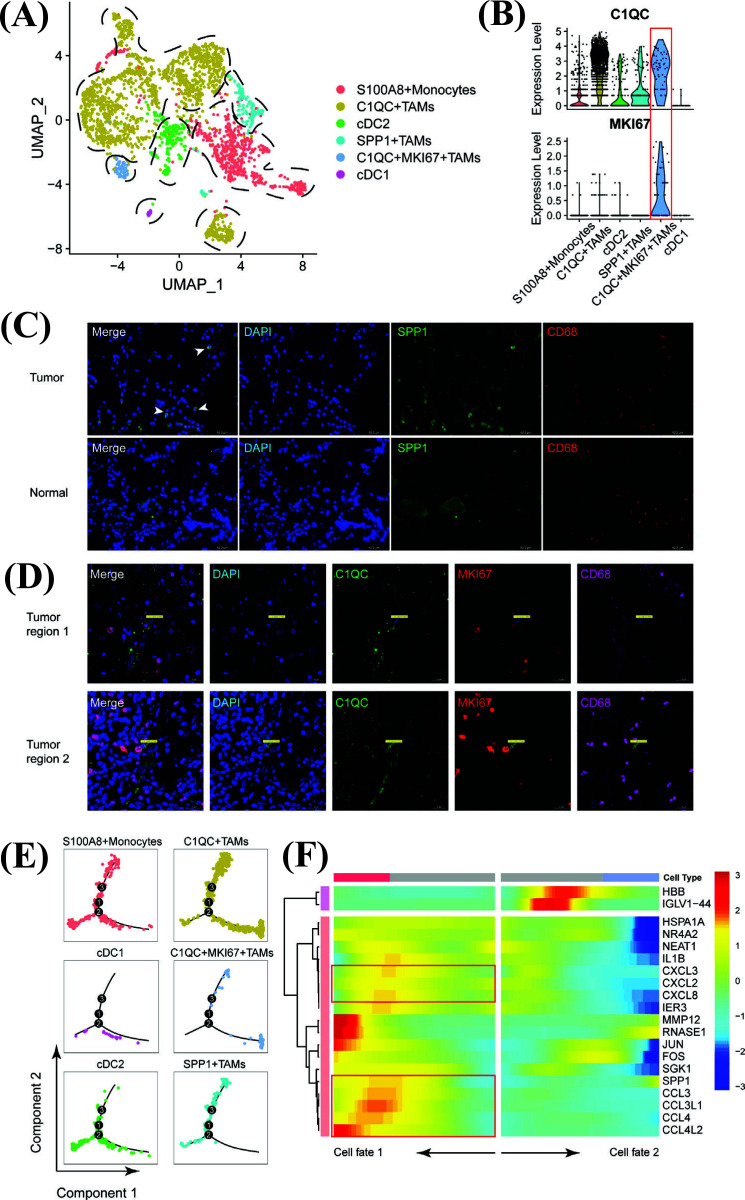
Characterization of scRNA-seq trace diverse myeloid cell lineages in GC. (**A**) UMAP plots of myeloid cells RNAseq profiled in this work. (**B**) Violin plots of C1QC and MKI67 expression in myeloid cell lineages. (**C**) IF assay confirmed the existence of specific macrophages in tumor tissues rather than adjacent normal tissues. (Scale bars: 62.2μm) (**D**) mIHC vertified the existence of C1QC^+^MKI67^+^TAMs within the tumor regions of GC patient, yellow boxes represent the C1QC^+^MKI67^+^TAMs. (Scale bars: 20μm) (**E**) Trajectories analysis of myeloid cells inferred by monocle 2 (Methods). (**F**) BEAM analysis on myeloid cell lineage branches shows the co-vary genes along the pseudotime trajectory.

Myeloid cells are complex cellular components of the TME and include macrophages, dendritic cells, and other cells in multiple distinct tumor types [44]. Tumor-infiltrating myeloid cells are critical for the suppression or activation of immune cells within TME. However, our understanding of the heterogeneous functions of myeloid cells in GC remains limited. Therefore, we analyzed the myeloid cells population and classified it into six subtypes (Fig. **4A**, Fig. **S5**, and Table **S7**): two DCs subpopulations were identified by highly expressed HLA-DRA and low expression of CD14, in detail, CD1C+ DCs were classified as cDC2, clusters expressed DAPP1 were cDC1 [44, 45]; cell subgroups highly expressed S100A8 and S100A9 were considered as S100A8^+^monocytes [21]; macrophages (CD68^high^, CD163^high^) were divided into three subgroups: C1QC^+^TAMs, SPP1^+^TAMs, and we found a proliferative C1QC^+^TAMs cluster also express MKI67^+^ then was defined as C1QC^+^MKI67^+^TAMs (Fig. **4B**) [21, 44]. To confirm these specific cell sublineages of macrophages in GC, we used IF and mIHC to identify the presence of SPP1^+^ TAMs and C1QC^+^MKI67^+^TAMs (Figs. **4C** and **D**). Pseudo-time analysis was utilized to infer the cell lineage relationships among these cells, which were divided into three branches and proved that C1QC^+^TAMs and C1QC^+^MKI67^+^TAMs originated from the same cell origins and had similar features (Fig. **4E**). Notably, SPP1^+^TAMs had similar origins to S100A8^+^monocytes (Fig. **4E**), and BEAM analysis demonstrated that SPP1, CCLs, and CXCLs co-varied with pseudotime (Fig. **4F**), suggesting that S100A8^+^monocytes recruited by tumor-derived chemokines can reprogram into SPP1^+^TAMs and perform pro-tumorigenesis functions. Future fundamental research should discern the key genes involved in monocyte reprogramming and develop novel drugs to block this process and inhibit the formation of intra-tumor suppressive immunity [46].

### 3.5 The Diversity of Cell Lineages Determines the Functional Heterogeneity of Myeloid Cells in GC

**Fig. (5):**
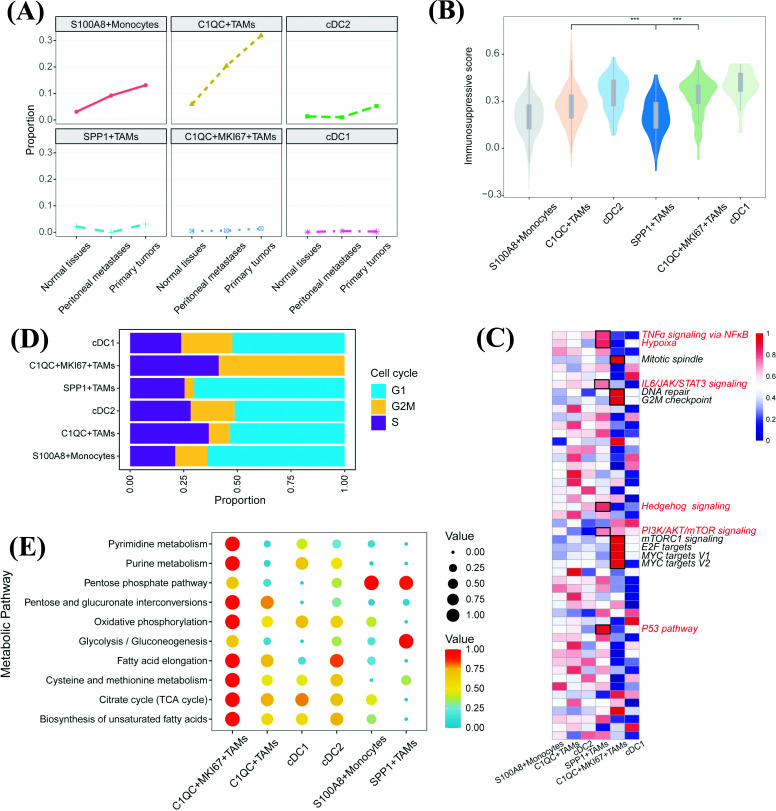
Diversity of cell lineages determine functional heterogeneity of myeloid cells in GC. (**A**) The line charts show the proportions of six tumor-infiltrating myeloid cell subsets in distinct tissue types. (**B**) Violin plot shows the immunosuppressed scores of six myeloid cells calculated by ssGSEA on known immunosuppressed gene set (Methods). (**C**) Heatmap shows ssGSEA result to explore the functional roles of six different myeloid cells. (**D**) Stacked-bar plot shows the cell cycle score among six myeloid cells. (**E**) Bubble plot shows the metabolic activities of distinct myeloid cell lineages calculated by scMetabolism algorithm. ***: *p*<0.05.

Given the heterogeneous myeloid cellular composition, a comprehensive functional analysis is helpful for elucidating its importance and providing value for clinical treatment. The different proportions of myeloid cells in different tissues revealed functional heterogeneity (Fig. **5A**). Most myeloid components were mainly present in the primary tumor tissue, of which the number of C1QC^+^TAMs was the highest in the primary tumor among all myeloid cells, followed by S100A8^+^monocytes, whereas these cells were scarce in the adjacent normal tissue. These differences hint at specific immune microenvironment atlases that exist in different tissues. Moreover, to illustrate the phenotypic differences in myeloid cells, we evaluated the pathway enrichment and immunosuppression scores [18] of these cells (Figs. **5B** and **C**, Table **S6** and **S8**). The results showed that C1QC^+^MKI67^+^TAMs had the highest activity in suppressing immunity compared to other TAMs (Fig. **5B**). Evaluation of known pathways with ssGSEA in myeloid cells revealed a strong expression of the cell cycle in C1QC^+^MKI67^+^TAMs; we specifically calculated the cell cycle scores of myeloid cells, and the analysis revealed that C1QC^+^MKI67^+^TAMs were subpopulations with high proliferation activity (Fig. **5D**). To confirm this, metabolic activities were analyzed, and as expected, we detected high nucleotide metabolic and glycometabolic levels in C1QC^+^MKI67^+^TAMs (Fig. **5E**, Table **S9**). The above analyses indicated that C1QC^+^MKI67^+^TAMs might be a portion of C1QC+TAMs with high proliferative activity and immunosuppressive functions. In addition, ssGSEA result indicated that SPP1^+^TAMs were related to hypoxia and some pro-tumor pathways: p53 pathway, IL6/JAK/STAT3 signaling, and TNFα/NF-κB pathway, *etc* (Fig. **5C**), suggesting a pro-tumorigenesis or pro-metastasis role in GC [44]. Our IF assay also suggested that SPP1^+^ TAMs could be found in tumor regions rather than in adjacent normal tissues, indicating their potential tumor-promoting function in patients with GC (Fig. **4C**).

### 3.6 Building CAFs-myeloid Cells-based Regulatory Network by Cellchat in Gastric Cancer

**Fig. (6):**
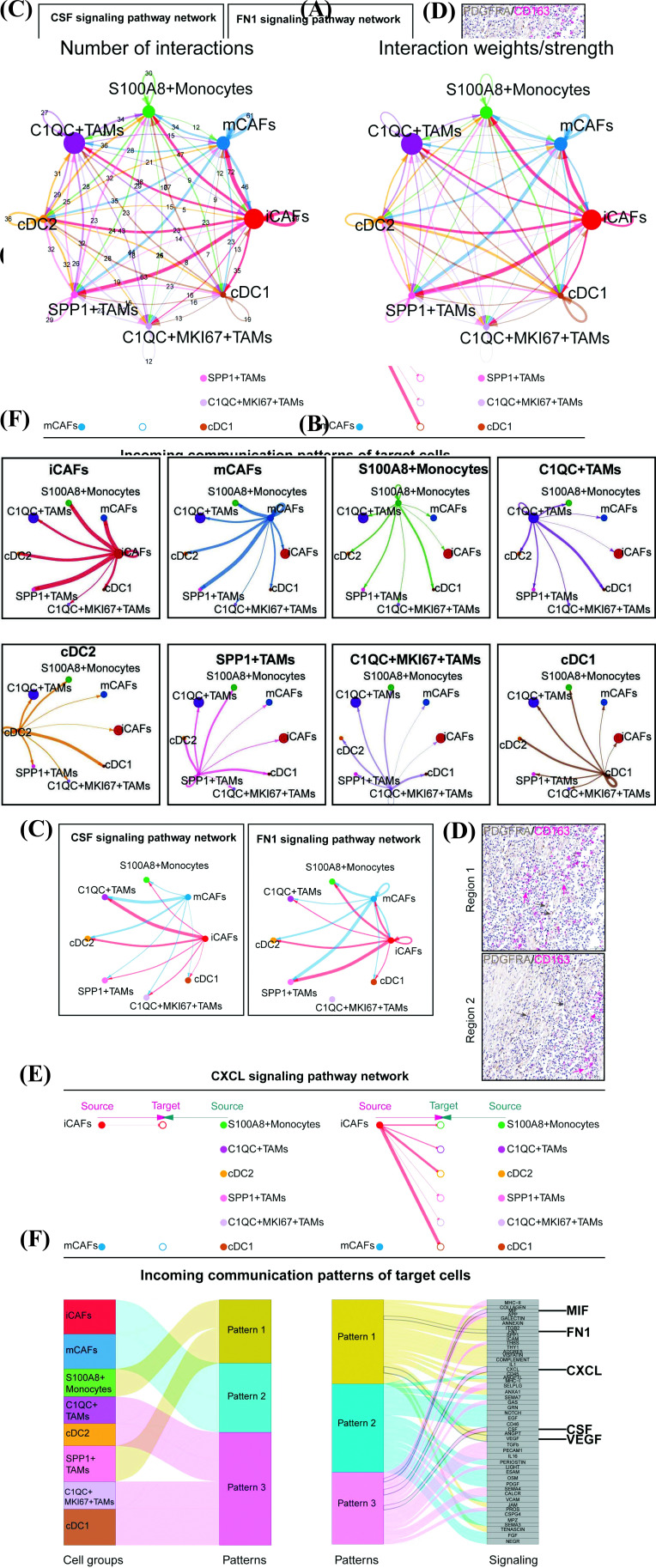
Building CAFs-myeloid cells based regulatory network by Cellchat in gastric cancer. (**A**) The circle plots show the overview of cell- cell interaction numbers and weights between CAFs and myeloid cells in all patients inferred by Cellchat (Methods): The plot shows the numbers of cell-cell interaction between CAFs and myeloid cells (left), The plot shows the weights of cell-cell interaction between CAFs and myeloid cells (right). (**B**) The circle plots exhibit the weights of cell-cell communications facet by cell types. (**C**) The circle plots of CSF and FN1 signaling pathway network inferred by Cellchat, the arrows represent the direction of interactions. (**D**) Immunohistochemical double-staining of PDGFRA/CD163 demonstrated the crosstalk between iCAFs and TAMs, brown arrows represent the iCAFs and pink arrows represent the TAMs (Scale bars: 50μm). (**E**) The Hierarchy plot of CXCL12 signaling pathway network reveals the unidirectional regulation of iCAFs towards distinct myeloid cells. (**F**) The sankey plot show the different incoming communication patterns of target cells in GC.

Emerging evidence shows that CAFs interact extensively with other immune components, including TAMs, which can promote cancer development through immune inhibition [9, 47]. Using CellChat, we explored cell-cell communication between the two subtypes of CAFs and the myeloid cells identified in our project. As shown in Figs. (**6A** and **B**), extensive crosstalk occurred between CAFs and myeloid cells, and iCAFs showed the highest communication with the other cell types. Notably, the dot plot showed significant ligand-receptor pair expression in these cells. Both two CAF subtypes expressed high levels of MIF, which could extensively interact with TAMs, except for C1QC+MKI67+TAMs, through the receptors CD74+CXCR4 or CD74+CD44 (Fig. **S6A**, Tables **S10**, and **S11**). MIF has been previously reported to be associated with the immunological escape caused by TAMs and macrophage polarization [48]. Therefore, we speculated that CAFs could improve the activities of TAMs and drive macrophages to M2 polarization through the MIF-CD74 axis, thus exerting immunosuppressive effects on the TME. We also suggest CAFs secreted CSF1 or FN1 could unidirectionally target cDC2, C1QC^+^TAMs, and SPP1^+^TAMs to promote macrophage or cDCs differentiation, as well as tumor development (Fig. **6C**) [44, 49-51]. Moreover, iCAFs could specifically generate CXCL12 to interact with tumor-infiltrating myeloid cells through the activation of the CXCL12/CXCR4 axis to induce the M2 polarization of macrophages or restrain the antigen presentation of cDCs to suppress antitumor immunity. We then applied immunohistochemical double-staining of PDGFRA/CD163 to demonstrate the crosstalk between iCAFs and TAMs in patients with GC (Figs. **6D** and **E**, Fig. **S6**) [52]. The incoming communication patterns of the target cells were analyzed to further improve our understanding of the communication patterns between these cells (Fig. **6F**). Notably, C1QC^+^ and SPP1^+^TAMs had different incoming communication patterns, underlining their functional differences. C1QC^+^TAMs mostly receive CSF, CXCL, and MIF signals from CAFs, while SPP1^+^TAMs can communicate with CAFs through the pro-tumor FN1 or VEGF pathways (Fig. **6F**). Combination therapies targeting these communication patterns may significantly inhibit the biological effects of macrophages in the TME. Collectively, our cell-cell interaction networks profile the complex crosstalk between CAFs and myeloid cells in the TME.

## 4 DISCUSSION

Currently, precision treatment has significantly reduced the mortality of patients with different tumor types [53, 54]. Classifying tumors into different molecular subtypes and target-specific tumor-driver genes has made some progress in patients with breast and colorectal cancers [55, 56]. However, precise therapies toward GC only received modest curative effects, of which extensive intra-tumor or intra-patient heterogeneity has been recognized as the major hurdle to the therapeutic effects of GC [2, 57]. Therefore, further studies should focus on tumor heterogeneity from a distinct perspective. Given the deeper understanding of the critical roles of the TME in tumorigenesis, metastasis, and chemoresistance, we are currently investigating the crucial factors of the TME and evaluating its clinical value [58-60]. Due to technological limitations in the past, the recognition of these factors in the TME remains nascent. Fast-emerging single-cell sequencing is a powerful tool that allows us to discern the TME and bridge the gap between basic research and clinical practice at the single-cell level [12, 61-63].

**Fig. (7):**
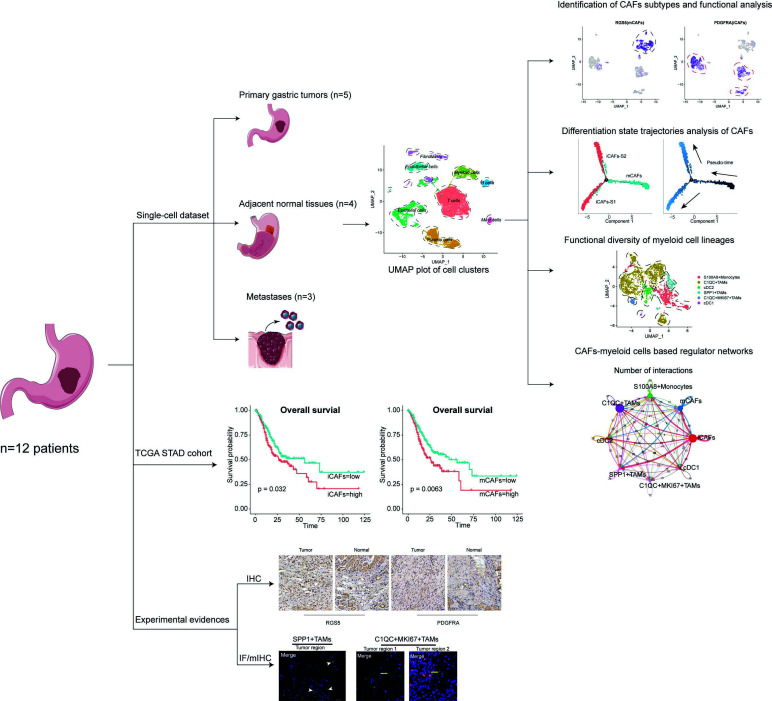
Workflow of the presenting project. Twelve samples were selected for our analysis including three different tissues types: primary tumors, adjacent tissues and metastases. Functions of CAFs subtypes and myeloid cells were fully investigated to discover the significance within tumor microenvironment. Besides, constructed regulator networks further detect the potential cell-cell communications between these types of cells in TME. TCGA STAD cohort was analyzed to confirm the clinical prognostic value of cancer-associated fibroblasts and myeloid cells in gastric cancer. In addition, fundamental experiments (IF, IHC, mIHC) were carried out to confirm the existence and functions of specific cell types identified in single cell dataset.

Here, we utilized single-cell RNA-seq to uncover the features of the TME across 31017 cells of three distinct GC tissue types, including primary tumors, adjacent normal tissues, and metastases (Fig. **7**). The intertissue functional heterogeneity of cell populations in the TME was comprehensively investigated. Eight distinct cell types were identified in this microenvironment. We suggest that a tissue-specific immune microenvironment exists in different GC tissue types by analyzing cell proportions and functional enrichment. Tumor regions reflect intensive immune inflammation, whereas metastases show extensive immunosuppression, which facilitates a premetastatic niche for metastatic cell survival [32, 64]. In addition, angiogenesis, hypoxic stimulus, and enhanced cell migration ability also accelerate the colonization of distant tissues by malignant cells [33], suggesting that the blockade of these biological processes may effectively restrain tumor development and metastasis in GC.

CAFs are heterogeneous stromal cell subgroups that accumulate in TME [65, 66]. Emerging evidence has identified different types of CAFs according to cell-specific signatures in multiple tumor types [34, 67-69]. Particularly, some studies have verified the existence of two specific CAFs named iCAFs and mCAFs in breast [70], pancreatic [35], and bladder carcinomas [29] by scRNA-seq. In this study, we verified the existence of iCAFs and mCAFs in GC using cell markers. Evidence has shown a pro-tumorigenic role for CAFs *via* metabolic crosstalk with other mesenchymal or tumor cells [37, 71, 72]. Characterization of the biological features fully displayed the metabolic pattern heterogeneity among different CAFs in GC, with special emphasis on the importance of iCAFs in lipid metabolism crosstalk within the microenvironment. In addition, GST family genes are responsible for CAF-mediated drug metabolism in metastases and could be possible targets for metastasis-related chemoresistance. The pivotal role of CAFs in the EMT process has been verified in previous studies [28, 73], and our research further showed that iCAFs, rather than mCAFs, preferentially express genes involved in EMT, suggesting that iCAFs might be promoting EMT, leading to tumor metastasis. In addition, two heterogeneous iCAF states were detected for the first time in our project based on pseudotime analysis. iCAFs-S1 and iCAFs-S2 possessed unique expression patterns in immune inflammation or ECM remodeling among different tissues, which was ascribed to the effects of tissue-specific niches. Stimuli of specific tumor microenvironments adjust the functional adaption of iCAFs to meet the needs of different tumor tissue progressions. Further research should be conducted to reveal the detailed mechanisms underlying the two iCAF states in distinct tissues for drug discovery.

Recent studies have revealed the complexity of myeloid cells in different tumor types [44, 74-76]. We identified six myeloid cell lineages with unique signatures in the present study. Differences in cell proportions among myeloid cell lineages reveal tissue-specific immune patterns in GC. Studies have reported that TAMs have multiple precursors, including monocytes and resident tissue macrophages (RTMs) [29, 44]. Our pseudo-time results also showed that the three macrophage subtypes, especially SPP1^+^TAMs, had similar origins as monocytes. The co-variation of SPP1 and chemokines along the pseudotime suggests that SPP1^+^TAMs are macrophages that might develop from monocytes that are recruited into tumor regions and undergo reprogramming *via* chemotaxis; therefore, targeting this biological process is expected to significantly repress the expansion and differentiation of TAMs, thus reducing the formation of a tumor-inhibiting microenvironment [44]. Functional enrichment also indicated that SPP1^+^TAMs are related to pro-tumorigenic pathways in the TME. The exploration of phenotypic heterogeneity in this study identified a novel cell sublineage named C1QC^+^MKI67^+^TAMs, which has the highest immunosuppression score and proliferative activity among TAMs, suggesting that this subgroup preferentially expresses genes involved in immune suppression and the cell cycle. Based on the above analyses, there are reasons to believe that these cells come from some C1QC^+^TAMs that have been activated by TME components with highly proliferative capability. Although Zhang *et al.* reported that C1QC^+^TAMs have a potential role in the activation of tumor immunity [44], our study further elucidated that highly proliferative C1QC^+^MKI67^+^TAMs have the opposite effect on immunoregulation, which shows the complexity of the tumor microenvironment.

Communication between various cellular components of the tumor microenvironment plays a key role in tumorigenesis and metastasis [5]. Although studies have focused on cell-cell crosstalk in the TME, considering the complexity of cell interactions and technical limitations, relevant research remains scarce and insufficient. To overcome these problems, we applied a novel algorithm (Cellchat) to infer the regulatory networks of cells using scRNA-seq [14]. CAFs and myeloid cells are abundantly existing tumor-stromal cells that contain various cell-cell interactions with other cells [6, 44]. Therefore, we built a regulator atlas based on ligand-receptor pairs of two types of cells and detected a pro-tumorigenic/pro-metastatic interplay between CAFs and myeloid cells in the TME. We underlined the value of CAFs in regulating tumor-promotion functions of myeloid cells through the production of MIF, CSF1, CXCL12, and FN1 [48, 50-52]. The incoming communication pattern analysis revealed a cell-cell interaction discrepancy between C1QC^+^ and SPP1^+^TAMs. Secreted cytokines (MIF/CSF1) mainly target C1QC^+^TAMs to impede the antitumor immune effects of TAMs rather than those of SPP1^+^TAMs, suggesting that anti-CSF or MIF treatments may be insufficient to deplete macrophage subgroups with tumor growth-promoting potential [44]. This phenomenon underlines the possible reasons for the poor efficacy of a single anti-CSF1 or MIF in cancer treatment. Multi-drug combined treatments, such as anti-CSF/FN1, may reverse resistance to monotherapy, which could be a promising strategy for single-drug-resistant patients.

Our study has some limitations. Single-cell sequencing was used to explore the crosstalk between distinct tumor microenvironment cells, including CAFs and myeloid cells. However, this technique could not completely reflect the interactions among different cells due to a lack of spatial information, and more techniques are required to improve the localization of single cells to compensate for the shortage of single-cell sequencing technology.

## CONCLUSION

In this study, we utilized single-cell sequencing technology to explore the tumor microenvironment heterogeneity in GC. We highlight the roles of CAFs and myeloid cells and provide an in-depth understanding of the cell-cell communication between these two cell types. In summary, this study provides a theoretical basis for future therapeutic strategies for GC.

## AUTHORS' CONTRIBUTIONS

PZW and HKW designed the study. PZW, RQF, and FC performed the experiments. PZW and TZW performed data analyses and visualization. PZW, RZH, and TZW were responsible for the quality control and interpretation of the results. PZW and HKW drafted the manuscript. All authors read, revised, and approved the final manuscript.

## Data Availability

The single-cell GC dataset of gastric cancer analyzed in the present study can be found in the GEO (GSE183904) repository: https://www.ncbi.nlm.nih.gov/geo/query/acc.cgi?acc=GSE183904. The GC transcriptome dataset was obtained from the TCGA STAD cohort at https://xenabrowser.net/datapages/.

## LIST OF ABBREVIATIONS

BEAM: Branched Expression Analysis Modeling
CAFs: Cancer-associated Fibroblasts
DAB: Diaminobenzidine
DCs: Dendritic Cells
DEGs: Differentially Expressed Genes
ECM: Extracellular Matrix Remodeling
EMT: Epithelial Mesenchymal Transition
GC: Gastric Cancer
GEO: Gene Expression Omnibus
GO: Gene Ontology
GSVA: Gene Set Variation Analysis
iCAFs: Inflammatory-cancer-associated Fibroblasts
IF: Immunofluorescence
IHC: Immunohistochemistry
mCAFs: Myo-cancer-associated Fibroblasts
mIHC: Multiplex Immunohistochemistry
NMF: Nonnegative Matrix Factorization
OS: Overall Survival
PCA: Principal Component Analysis
scRNA-seq: Single-cell RNA Sequencing
ssGSEA: Single Sample Gene Set Enrichment Analysis
TAMs: Tumor-associated Macrophages
TCGA: The Cancer Genome Atlas
TME: Tumor Microenvironment
UMAP: Uniform Manifold Approximation and Projection
