# Supplementary material for: Crosstalk Between Cancer-associated Fibroblasts and Myeloid Cells Shapes the Heterogeneous Microenvironment of Gastric Cancer
Source: Curr Genomics. 2024 Jun 11;25(5):390–411. doi: 10.2174/0113892029300608240531111743 (PMC11420565; doi:10.2174/0113892029300608240531111743)
Supplement: Supplementary file 1 [file CG-25-390_SD1.zip › CG-25-390/6b-BMS-CG-2023-235 Supplementary Material.pdf]

Supplementary Material

Crosstalk Between Cancer-associated Fibroblasts and Myeloid Cells Shapes the Heterogeneous Microenvironment of Gastric Cancer

Zhiwei Peng[1], Can Fang[1], Zhiwei Tong[1], Qiufan Rao[2], Zihao Ren[1] and Kongwang Hu[1,3,*]

[1]Department of General Surgery, First Affiliated Hospital of Anhui Medical University, Hefei, Anhui 230022, China; [2]Anhui Province Key Laboratory of Major Autoimmune Diseases, Anhui Institute of Innovative Drugs, School of Pharmacy, Anhui Medical University, Hefei, Anhui 230032, China; [3]Department of General Surgery, Fuyang Affiliated Hospital of Anhui Medical University, Fuyang, Anhui 236000, China

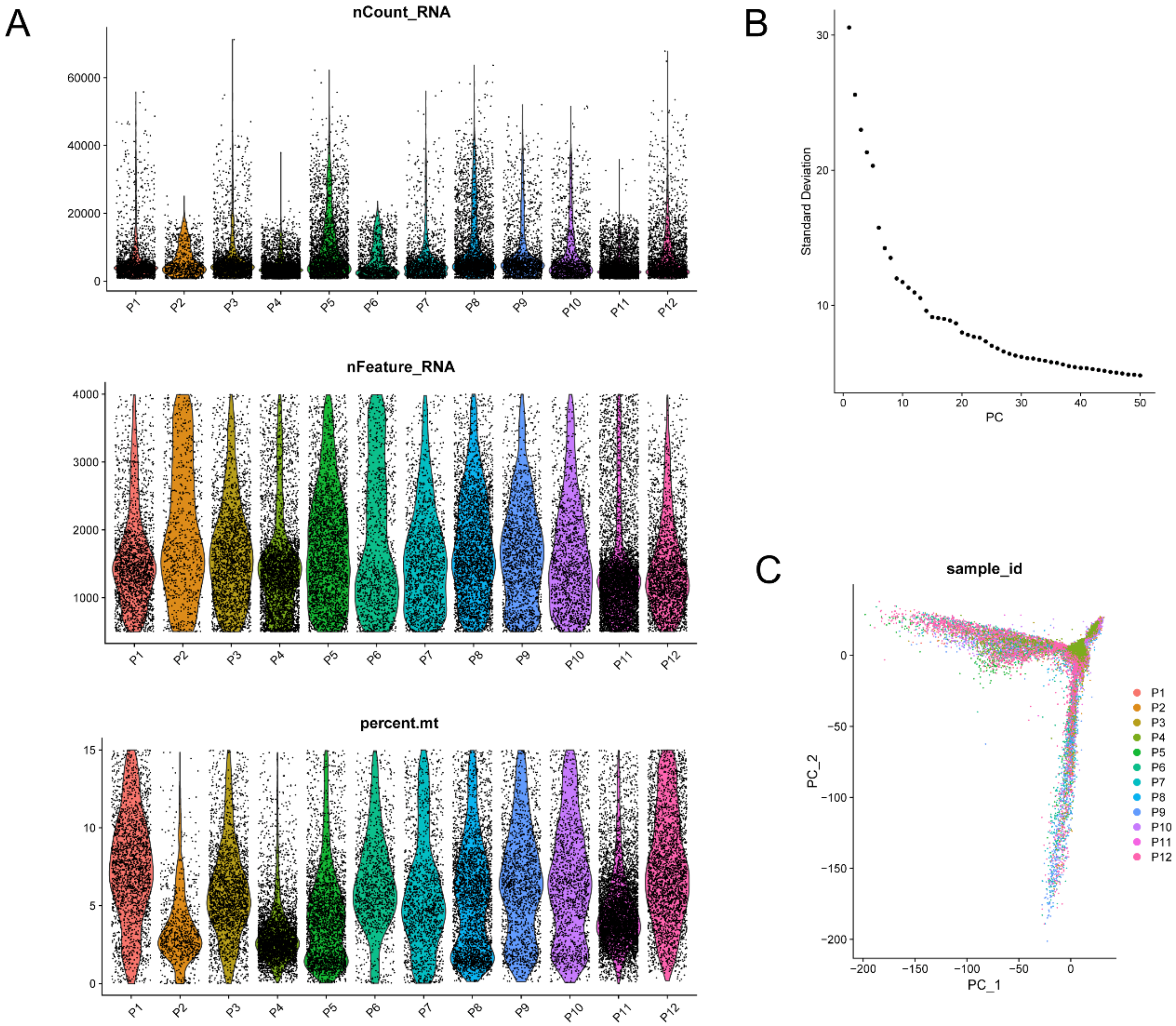

Fig. S1. The results of data quality of single-cell dataset analyzed in our project. (A) Violin plots show the RNA features, RNA counts and mitochondrial percentages after quality control. (B) The elbow plot is applied to determine the numbers of principal component. (C) PCA plot of twelve samples.

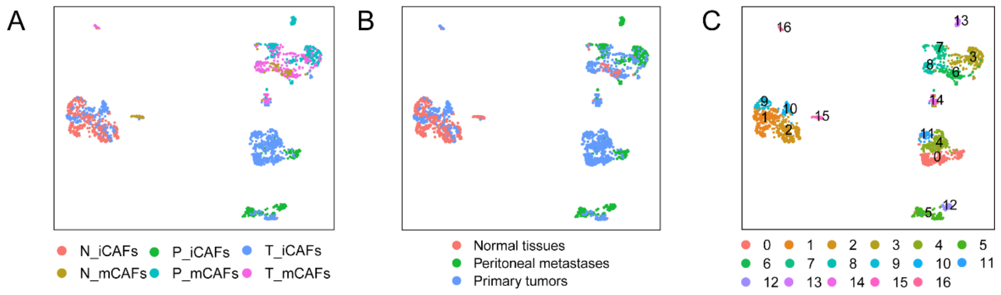

Fig. S2. UMAP plots of single cell RNA-seq of fibroblasts profiled in this project colored by cell types (A), tissue types (B) and clusters (C).

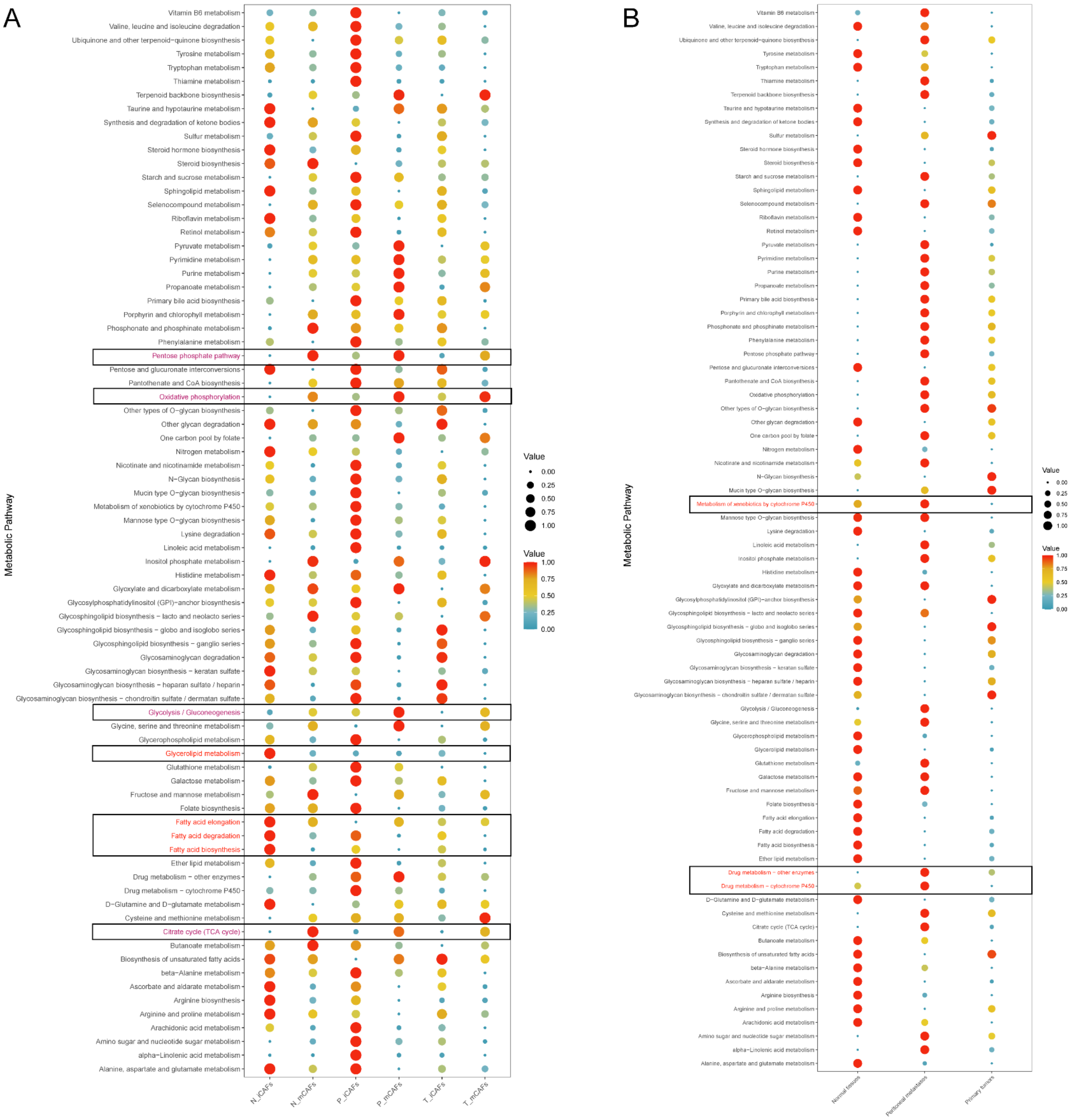

Fig S3. Dot plots show the metabolic activity of CAFs inferred by scMetabolism. (A) Dot plot shows metabolic activity of different CAFs subtypes. (B) Dot plot shows CAFs-mediated tissue-specific metabolic patterns. (Significantly different metabolic patterns are marked with black boxes.)

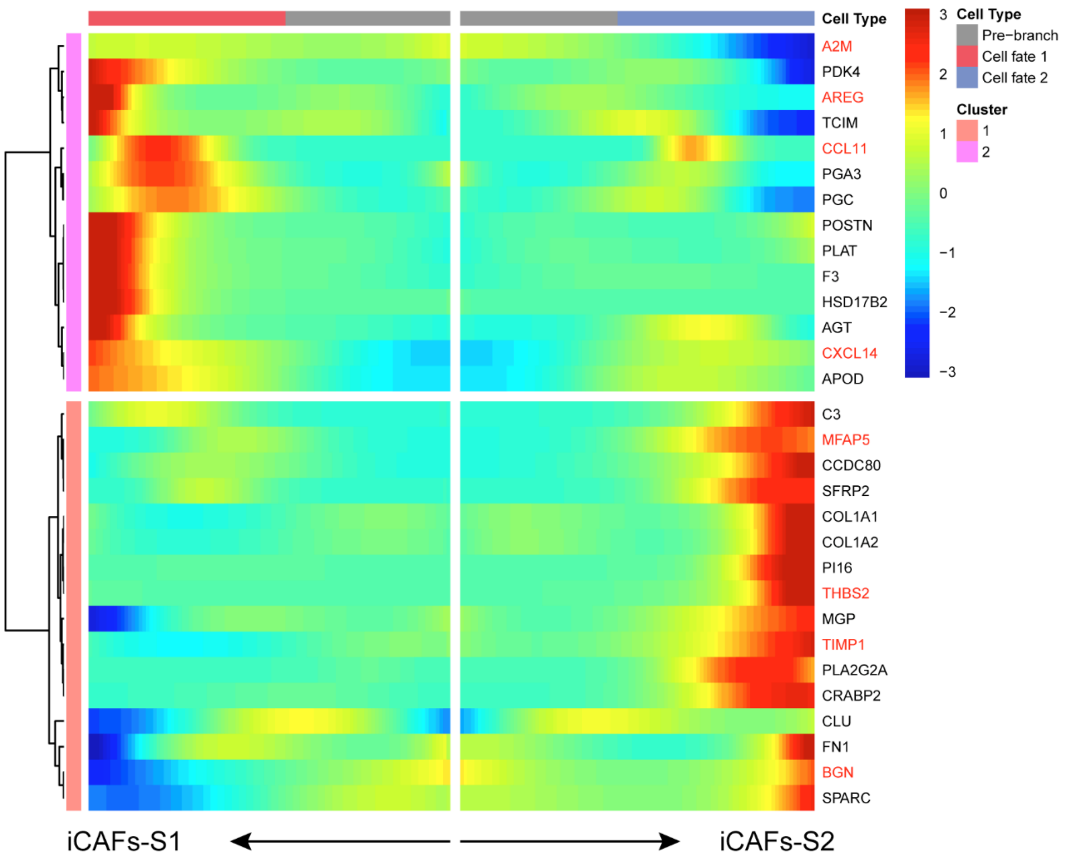

Fig S4. BEAM analysis on DEGs of two substates of iCAFs. Heatmap shows the differentially expressed genes involved in two states of iCAFs. (Genes colored with red marks are DEGs with specific functions further analysis in our project.)

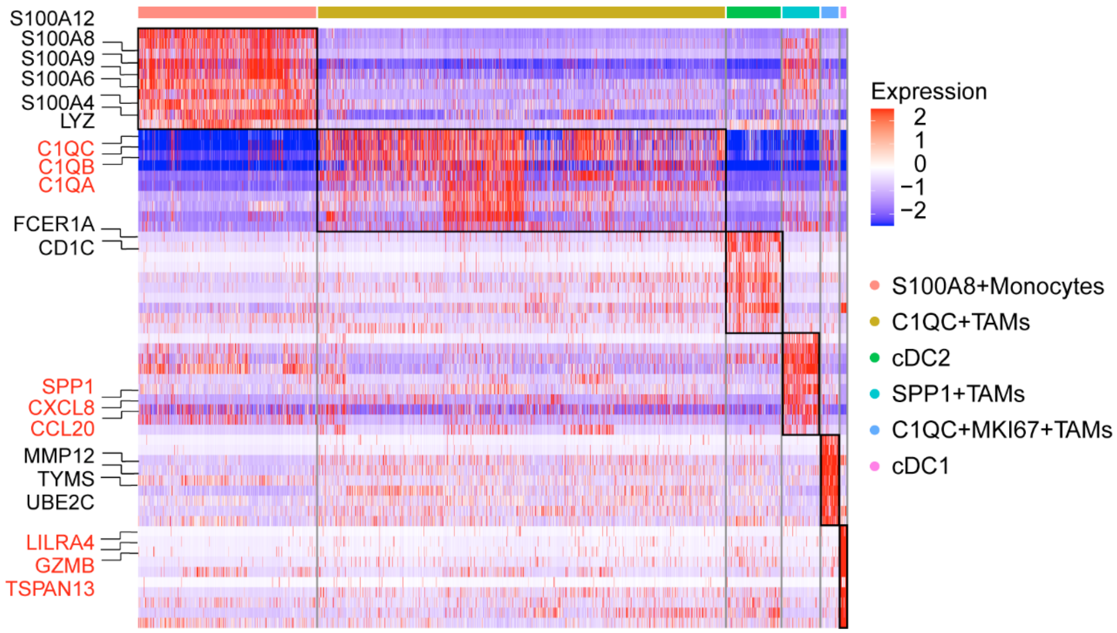

Fig S5. Heatmap of top 10 high variable genes expression in each myeloid cell cluster.

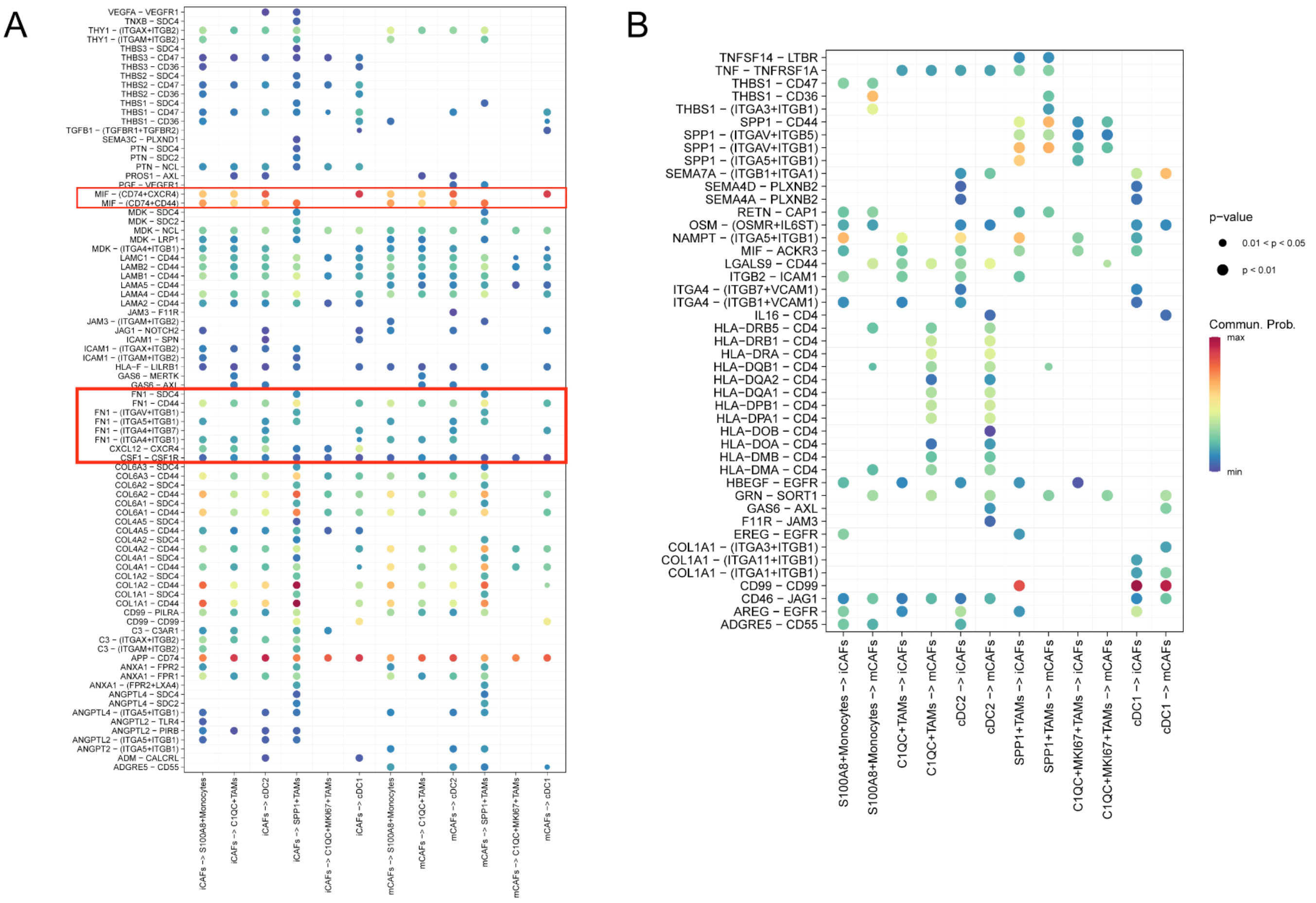

Fig S6. Bubble plots show the ligand-receptor pairs expression between CAFs and myeloid cells inferred by Cellchat. (A) Bubble plot shows the regulatory networks of CAFs targeting myeloid cells. (B) Bubble plot shows the regulatory networks of myeloid cells targeting CAFs. (Significant ligand-receptor pairs were highlighted with red markers.)
